# Improving reproducibility by using high-throughput observational studies with empirical calibration

**DOI:** 10.1098/rsta.2017.0356

**Published:** 2018-08-06

**Authors:** Martijn J. Schuemie, Patrick B. Ryan, George Hripcsak, David Madigan, Marc A. Suchard

**Affiliations:** 1 Observational Health Data Sciences and Informatics (OHDSI), New York, NY 10032, USA; 2 Epidemiology Analytics, Janssen Research and Development, Titusville, NJ 08560, USA; 3 Department of Biomedical Informatics, Columbia University Medical Center, New York, NY 10032, USA; 4 Medical Informatics Services, New York-Presbyterian Hospital, New York, NY 10032, USA; 5 Department of Statistics, Columbia University, New York, NY 10027, USA; 6 Department of Biomathematics, University of California, Los Angeles, CA 90095, USA; 7 Department of Biostatistics, University of California, Los Angeles, CA 90095, USA; 8 Department of Human Genetics, University of California, Los Angeles, CA 90095, USA

**Keywords:** observational research, reproducibility, publication bias, medicine

## Abstract

Concerns over reproducibility in science extend to research using existing healthcare data; many observational studies investigating the same topic produce conflicting results, even when using the same data. To address this problem, we propose a paradigm shift. The current paradigm centres on generating one estimate at a time using a unique study design with unknown reliability and publishing (or not) one estimate at a time. The new paradigm advocates for high-throughput observational studies using consistent and standardized methods, allowing evaluation, calibration and unbiased dissemination to generate a more reliable and complete evidence base. We demonstrate this new paradigm by comparing all depression treatments for a set of outcomes, producing 17 718 hazard ratios, each using methodology on par with current best practice. We furthermore include control hypotheses to evaluate and calibrate our evidence generation process. Results show good transitivity and consistency between databases, and agree with four out of the five findings from clinical trials. The distribution of effect size estimates reported in the literature reveals an absence of small or null effects, with a sharp cut-off at *p* = 0.05. No such phenomena were observed in our results, suggesting more complete and more reliable evidence.

This article is part of a discussion meeting issue ‘The growing ubiquity of algorithms in society: implications, impacts and innovations’.

## 1. Introduction

Great concern exists over reproducibility in science, with many scientists even using the term ‘reproducibility crisis’ [1]. Low sample size, small effect sizes, data dredging (including p-hacking), conflicts of interest, large numbers of scientists working competitively in silos without combining their efforts and so on may conspire to dramatically increase the probability that a published finding is incorrect [2]. Although many solutions have been proposed, including pre-registering studies, open science, team research and better reporting, adoption of these solutions is still lacking [3,4]. Here, we focus on reproducibility in observational research using large-scale databases of existing health records, where we believe a complementary solution is viable that would vastly improve reproducibility, while at the same time generate large amounts of reliable scientific evidence. This approach is most useful when multiple such databases are available, but might also be used in a single large database.

Existing healthcare data, such as claims and electronic health records, hold the promise of providing new insights to improve patient care. These data capture details of real-world experiences of patients and their encounters with the healthcare system, allowing the study of many types of therapies and revealing benefits received and harm done. Certainly, there exist limits to the range of questions that these data can answer as they are based on interactions with the healthcare system and depend on accurate recording of events. There is also an information asymmetry as ‘harms’ tend to come to medical attention and are easily reflected in these data while ‘benefits’ are often neither easily reflected in these data nor do they tend to drive patients to clinical encounters. Observational studies are more susceptible to bias, placing them lower in the hierarchy of clinical evidence than randomized clinical trials (RCTs). Nonetheless, these data could yield a wealth of insights that go well beyond what can be explored through other sources of evidence.

### (a) A new paradigm

**Figure 1.**
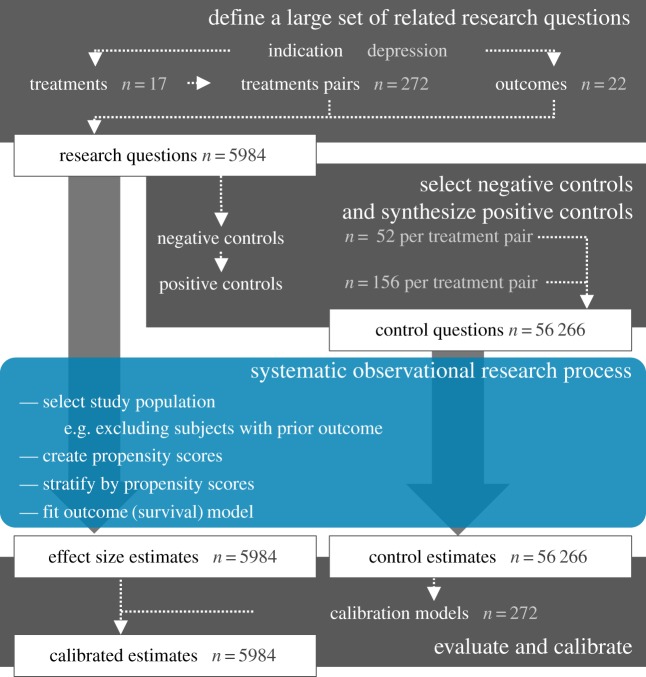
High-throughput observational study design with empirical calibration, applied to the comparison of depression treatments. We apply this design to four large insurance claims databases. (Online version in colour.)

Current observational research relies on one-off studies answering one question at a time with unique methodology and therefore unknown reliability, and disseminating these results (or not) one estimate at a time. Here, we propose to unlock the potential of existing healthcare data by defining a high-throughput approach to observational research; we systematically compare all treatments for a given indication for a large set of outcomes captured in data from the Observational Health Data Science and Informatics (OHDSI) [5] research network. We adjust for measured confounders using propensity score stratification, a commonly used confounder-adjustment strategy, but instead of the current practice of hand-picking covariates for the propensity model, we employ a completely data-driven approach to variable selection. In addition, uncertainty due to residual observational study bias, for example due to unmeasured confounders, is quantified by using control hypotheses (research questions with known answers). We employ both real negative control hypotheses (where the true hazard ratio is known to be 1) as well as synthetic positive control hypotheses (where the true hazard ratio is of known magnitude greater than 1), created by modifying negative controls. We subsequently express the observed uncertainty due to residual bias in calibrated confidence intervals (CIs), ensuring, for example, that for 95% of our negative and positive controls, the 95% calibrated CI contains the true hazard ratio [6]. We disseminate all results, thereby not only providing evidence at large scale, but also preventing publication bias. We demonstrate this new paradigm by comparing all treatments for depression for a large set of health outcomes using four large insurance claims databases, as depicted in figure 1. We evaluate our results in terms of transitivity and between-database consistency, and agreement with effects known from clinical trials. We also show that our distribution of estimates is markedly different from the distribution observed in the literature, suggesting more complete and more reliable evidence.

## 2. Material and methods

### (a) Comparison of depression treatments

As an example of our proposed high-throughput observational research, we focus on the risk of specific outcomes across treatment choices for major depressive disorder. Depression is the leading cause of disability worldwide, affecting an estimated 350 million people globally [7], with multiple pharmacological and non-pharmacological treatments from which to choose. We identified 17 depression treatments to compare, and 22 outcomes of clinical interest (table 1). As a result, we have 17 × (17 − 1) × 22 = 5984 research questions.

**Table 1.** Treatments and outcomes of interest.

treatments of interest	outcomes of interest
amitriptyline	acute liver injury
bupropion	acute myocardial infarction
citalopram	alopecia
desvenlafaxine	constipation
duloxetine	decreased libido
electroconvulsive therapy	delirium
escitalopram	diarrhoea
fluoxetine	fracture
mirtazapine	gastrointestinal haemorrhage
paroxetine	hyperprolactinaemia
psychotherapy	hyponatraemia
sertraline	hypotension
trazodone	hypothyroidism
venlafaxine	insomnia
vilazodone	nausea
	open-angle glaucoma
	seizure
	stroke
	suicide and suicidal ideation
	tinnitus
	vent. arr. and sudden cardiac death
	vertigo

Our study follows a typical comparative effectiveness design [8], comparing a target treatment (T) to a comparator treatment (C) for the risk of an outcome (O). We create definitions of all Ts, Cs and Os listed in table 1, based on clinical knowledge and our understanding of the databases (see the electronic supplementary material), and pre-specify the rules by which these definitions should be adapted for any specific combination of T, C and O. For example, T and C are restricted to the calendar time when both treatments were recorded in the database, and people with prior O are removed from both T and C. Because of the observational nature of the study, subjects in T may differ from subjects in C in ways that could bias effect estimation. We apply a commonly used confounding adjustment strategy—stratification by propensity scores—to make the two cohorts more comparable. We define time-at-risk to start on the day of treatment initiation and stop when treatment stops, allowing for a 30 day gap in treatment continuation. We specify a sensitivity analysis where the time-at-risk is defined to stop at end of observation (end of enrolment or end of study period, whichever comes first). Hazard ratios are estimated using a Cox proportional model conditioned on the propensity score strata.

The full source code for executing this study is available online (https://github.com/OHDSI/StudyProtocols/tree/master/LargeScalePopEst). Execution time greatly depends on the size of the data. Producing the results reported in this paper took five weeks on a computer with 32 processing cores and 168 GB of memory.

### (b) Propensity score stratification

Adjustment for baseline confounders is done by fitting a propensity model and creating propensity scores for every pair of exposures. The propensity score is the probability of a subject receiving one treatment instead of the other, conditional on baseline characteristics [9]. We create a data-driven process that entertains a large set of predefined baseline covariates—often tens of thousands—consistently for all combinations of T, C and O, and use the data to decide which combination of these characteristics are most predictive of the treatment assignment.

The following variables are included in all propensity models:

— demographics (age in 5 year increments, gender, race, ethnicity, year of index date, month of index date),
— condition occurrence (one or more variables per diagnose code),
— condition era (one or more variables per diagnose code),
— condition group (one or more variables per MedDRA group or SNOMED groups),
— drug exposure (one or more variables per drug code),
— drug era (one or more variables per RxNorm ingredient),
— drug group (one or more variables per ATC group),
— procedure occurrence (one or more variables per procedure code),
— observations (one or more variables per observation concept ID),
— measurements (one or more variables per measurement concept ID, including variables for within/above/below normal range),
— risk scores (including Charlson, DCSI, CHADS2, CHADS2VASc).

Variables with fewer than 100 non-zero values are discarded because these are unlikely to receive non-zero coefficients after regularization, and excluding them reduces the computational burden. For full details on the covariates used in our models, refer to the FeatureExtraction package (https://github.com/OHDSI/FeatureExtraction).

The propensity models are fitted using L_1_ regularized regression [10], using 10-fold cross-validation to select the regularization parameter. These propensity scores are used to stratify the target and comparator cohorts in 10 strata, and the proportional hazards outcome models are conditioned on the strata [9].

### (c) Control hypotheses

We evaluate our process by applying it to research hypotheses where the truth is known with a high degree of certainty. Such a gold standard should include both negative and positive controls.

For comparative effectiveness studies, we define negative controls as TCO combinations where neither T nor C causes O and where therefore the true hazard ratio is equal to one. In practice, we identify negative controls by selecting exposures and outcomes that are well studied, but for which no evidence in the literature or elsewhere suggests a relationship. For example, one negative control outcome is ‘ingrown nail’, because we firmly believe that no depression treatment causes ingrown nails. It is important to note that although there is no causal relationship, some antidepressants may be associated with ingrown nails, for example, because the treatment is prescribed primarily for the elderly, where this condition is more prevalent. This allows us to test whether our confounding adjustment can correct for this confounding association, and produces estimates consistent with the null.

A candidate list of negative control outcomes was generated by identifying outcomes with no evidence of being causally related to any exposure of interest [11]. This evidence was searched in the literature through MeSH headings [12] and natural language processing [13], spontaneous reports of adverse events [14] and product labels in the USA [15] and Europe [16]. The candidate outcomes were then reverse sorted by prevalence in the observational databases and manually curated until a reasonably sized set of negative controls was established. The final list of 52 negative control outcomes is provided in table 2.

**Table 2.** Negative control outcomes. Outcomes not believed to be caused by any of the exposures of interest.

acariasis	ingrowing nail
amyloidosis	iridocyclitis
ankylosing spondylitis	irritable bowel syndrome
aseptic necrosis of bone	lesion of cervix
astigmatism	Lyme disease
Bell's palsy	malignant neoplasm of endocrine gland
benign epithelial neoplasm of skin	nononeuropathy
chalazion	onychomycosis
chondromalacia	osteochondropathy
Crohn's disease	paraplegia
croup	polyp of intestine
diabetic oculopathy	presbyopia
endocarditis	pulmonary tuberculosis
endometrial hyperplasia	rectal mass
enthesopathy	sarcoidosis
epicondylitis	scar
Epstein–Barr virus disease	seborrhoeic keratosis
fracture of upper limb	septic shock
gallstone	Sjogren's syndrome
genital herpes simplex	Tietze's disease
haemangioma	tonsillitis
Hodgkin's disease	toxic goitre
human papilloma virus infection	ulcerative colitis
hypoglycaemic coma	viral conjunctivitis
hypopituitarism	viral hepatitis
impetigo	visceroptosis

Positive controls in this case are outcomes believed to be caused by one exposure, but not the other. Unfortunately, real positive controls for observational research tend to be problematic for three reasons: first, when comparing the effect of two treatments, there often is a paucity of positive controls relevant for that specific comparison. Second, even if positive controls are available, the magnitude of the effect size may not be known with great accuracy, and often depends on the population in which it is measured. Third, when treatments are widely known to cause a particular outcome, this will shape the behaviour of physicians prescribing the treatment, for example by taking actions to mitigate the risk of unwanted outcomes, thereby rendering the positive controls useless as a means for evaluation [17]. We therefore use synthetic positive controls [6], created by modifying a negative control through injection of additional, simulated occurrences of the outcome. To preserve (measured) confounding, simulated outcome occurrences are sampled from the probability distribution derived from a predictive model fitted on the data. These models use the same covariates as the propensity models as independent variables, and the occurrence of the negative control outcomes as the dependent variables. Target true hazard ratios for the positive control synthesis are 1.5, 2 and 4, so using the 52 negative controls, we are able to construct 52 × 3 = 156 positive control outcomes for every comparison of two treatments. No negative control outcome model is fitted and no positive controls are created if there were fewer than 100 persons with the outcome across all exposures, because below this threshold fitted models are likely to have only coefficients of zero (except the intercept). No injection is performed if, for the exposure that is considered for injection, there were fewer than 25 persons with the outcome before injection, to avoid rounding errors in the true effect size.

With a gold standard in place, we evaluate whether our process produces results in line with the gold standard effect sizes. Importantly, we estimate CI coverage probability—the proportion of time that the CI contains the true value of interest. For example, we expect a 95% CI to cover the truth 95% of the time. We also apply a calibration procedure described elsewhere [6] that attempts to restore nominal coverage by adjusting the CIs, similarly to how one would calibrate a scale by using objects of known weight. In short, this procedure first estimates the distribution of systematic error using the observed estimates for negative and positive controls. We assume this distribution is Gaussian with a mean and log standard deviation linearly related to the true effect size. Using the estimated distribution, we then generate calibrated CIs considering both random and systematic error. Typically, but not necessarily, the calibrated CI is wider than the nominal CI, reflecting the problems unaccounted for in the standard procedure (such as unmeasured confounding, selection bias and measurement error) but accounted for in the calibration.

### (d) Observational databases

The analyses have been performed across a network of observational healthcare databases. All databases have been transformed into the OMOP Common Data Model, version 5. The complete specification for OMOP Common Data Model, version 5, is available at: https://github.com/OHDSI/CommonDataModel. The following databases have been included in this analysis:

— Truven MarketScan Commercial Claims and Encounters (CCAE),
— Truven MarketScan Medicare Supplemental Beneficiaries (MDCR),
— Truven MarketScan Multi-state Medicaid (MDCD),
— OptumInsight's de-identified Clinformatics™ Datamart (Optum).

### (e) Truven MarketScan Commercial Claims and Encounters

CCAE is an administrative health claims database for active employees, early retirees, COBRA continues and their dependents insured by employer-sponsored plans (individuals in plans or product lines with fee-for-service plans and fully capitated or partially capitated plans). As of 1 November 2016, CCAE contained 131 million patients with patient-level observations from January 2000 through July 2016. Source codes used in CCAE include: conditions: ICD-9-CM; drugs: NDC, HCPCS, ICD-9-CM; procedures: CPT-4, HCPCS, ICD-9-CM; laboratory: LOINC.

The ETL specification for transforming CCAE into the OMOP CDM is available at https://github.com/OHDSI/ETL-CDMBuilder/tree/master/man/TRUVEN_CCAE_MDCR.

### (f) Truven MarketScan Medicare Supplemental Beneficiaries

MDCR is an administrative health claims database for Medicare-eligible active and retired employees and their Medicare-eligible dependents from employer-sponsored supplemental plans (predominantly fee-for-service plans). Only plans where both the Medicare-paid amounts and the employer-paid amounts were available and evident on the claims were selected for this database. As of 1 November 2016, MDCR contained 9.6 million patients with patient-level observations from January 2000 through July 2016. Source codes used in MDCR include: conditions: ICD-9-CM; drugs: NDC, HCPCS, ICD-9-CM; procedures: CPT-4, HCPCS, ICD-9-CM; laboratory: LOINC.

The ETL specification for transforming MDCR into the OMOP CDM is available at https://github.com/OHDSI/ETL-CDMBuilder/tree/master/man/TRUVEN_CCAE_MDCR.

### (g) Truven MarketScan Multi-state Medicaid

MDCD is an administrative health claims database for the pooled healthcare experience of Medicaid enrolees from multiple states. As of 1 November 2016, MDCD contained 21.6 million patients with patient-level observations from January 2006 through December 2014. Source codes used in MDCD include: conditions: ICD-9-CM; drugs: NDC, HCPCS, ICD-9-CM; procedures: CPT-4, HCPCS, ICD-9-CM; laboratory: LOINC.

The ETL specification for transforming MDCD into the OMOP CDM is available at https://github.com/OHDSI/ETL-CDMBuilder/tree/master/man/TRUVEN_MDCD.

### (h) OptumInsight's de-identified Clinformatics Datamart (Optum)

OptumInsight's de-identified Clinformatics Datamart (Eden Prairie, MN, USA) is an administrative health claims database for members of United Healthcare who enrolled in commercial plans (including ASO, 36.31 M), Medicaid (prior to July 2010, 1.25 M) and Legacy Medicare Choice (prior to January 2006, 0.36 M) with both medical and prescription drug coverage. As of 1 November 2016, Optum contained 74.7 million patients with patient-level observations from June 2000 through June 2016. Source codes used in Optum include: conditions: ICD-9-CM; drugs: NDC, HCPCS, ICD-9-CM; procedures: CPT-4, HCPCS, ICD-9-CM; laboratory: LOINC.

The ETL specification for transforming Optum into the OMOP CDM is available at https://github.com/OHDSI/ETL-CDMBuilder/tree/master/man/OPTUM_EXTENDED.

### (i) Extraction from the literature

Citations of observational studies were identified in PubMed using the following query:

("population-based" [Title/Abstract] OR observational [Title/Abstract] OR pharmacoepidemiology [Title/Abstract]) AND (("Cohort Studies" [MeSH] OR "cohort" [Title/Abstract] OR "propensity score" [Title/Abstract]) OR ("Case-Control Studies" [MeSH] OR "case control" [Title/Abstract]) OR ("self controlled case series" [Title/Abstract] OR ("sccs" [Title/Abstract] AND "self-controlled" [Title/Abstract])) OR ("case-crossover" [Title/Abstract]) ) AND ("1900/01/01"[PDAT]:"3000/12/31"[PDAT])

In total, 102 874 citations were retrieved. The abstracts were automatically scanned for occurrences of the following regular expression:

"("+emPattern+" ?\\(|\\([^)]*"+emPattern+")[^(]*("+pValuePattern+"|"+ciPattern+")[^(]*\\)"

where

numberPattern = "[0–9][0–9]?[0–9]?\\.[0–9][0–9]?[0–9]?"

emPattern = "(odds ratio|o.r.|or|relative risk|r.r.|rr|hazard ratio|h.r.|hr|hazard|rate ratio)([^0-9a-z]*| is | of )"+numberPattern

pValuePattern = "p ?[<=>] ?0?\\.[0–9][0–9]?[0–9]?"

ciPattern = numberPattern+" ?(-|to|,) ?" +numberPattern

In total, 59 196 estimates were found in 24 027 abstracts. The standard error was computed from either the CI or *p*-value that was found in combination with an effect size estimate. If both a *p*-value and CI were present, the CI was used. The full list of estimates is provided in the electronic supplementary material, Data S2. To remove visual artefacts due to rounding, for visualization purposes only, random noise was added to the estimates, CIs and *p*-values, so that the noisy numbers would still round to the numbers reported in the abstracts. For example, a hazard ratio of 1.5 was converted to a random number between 1.450000001 and 1.549999999.

A subset of articles related to depression treatment was identified using the PubMed query:

(depression OR antidepressant) AND ("serotonin reuptake inhibitors" OR "tricyclic antidepressant" OR Bupropion OR Mirtazapine OR Trazodone OR Desvenlafaxine OR duloxetine OR venlafaxine OR Citalopram OR Escitalopram OR Fluoxetine OR Paroxetine OR Sertraline OR vilazodone OR Amitriptyline OR Doxepin OR Nortriptyline or psychotherapy or "electroconvulsive therapy").

## 3. Results

### (a) Example single research hypothesis

**Figure 2.**
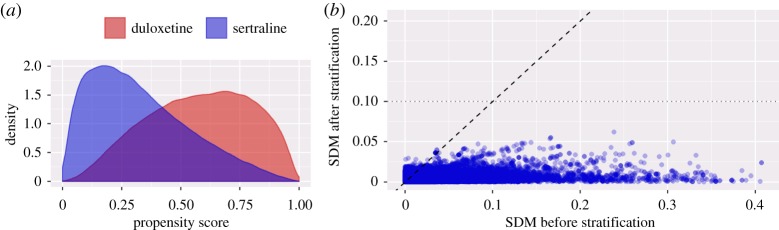
Cohort comparability and balance for duloxetine versus sertraline new users from the CCAE database. (*a*) Propensity score distributions for each cohort. (*b*) Absolute values of the standardized difference of the mean (SDM) before and after stratification for the 59 038 covariates established at baseline.

We demonstrate our high-throughput process by first showing the analysis for a single research question: the comparison of duloxetine to sertraline for the risk of stroke, using the Truven MarketScan Commercial Claims and Encounters (CCAE) database. We compare our approach to a previously published study by Lee *et al*. [18]. Whereas that study compares new users of the entire drug classes to which these drugs belong, our analysis investigates new users of the two specific drugs. Both Lee *et al*. and our analysis require 12 months of continuous observation prior to treatment initiation, exclude people exposed to both drugs and people with prior strokes and use stratification on the propensity score to address confounding. Follow-up is defined as starting on the day of treatment initiation and stopping on the day of the outcome, discontinuation of treatment (allowing a 30-day gap between treatments), or disenrollment. Lee *et al*. hand-picked 74 covariates such as age, sex and various selected drugs and diagnoses to create a propensity model. By contrast, we used a data-driven approach to generate a propensity model based on 59 038 covariates. Figure 2*a* shows our propensity score distribution across new users.

For many subjects, treatment assignment is highly dependent upon their baseline characteristics, indicating that the groups are fundamentally different and that without adjustment, there is a high likelihood of confounding. On the other hand, figure 2*a* also reveals substantial overlap, implying that propensity score adjustment should be able to make the groups equivalent, at least with regard to measured covariates. Indeed, figure 2*b* shows that many covariates are imbalanced prior to adjustment, but after stratification, all covariates have a standardized difference of mean smaller than 0.1, generally assumed to indicate adequate balance. This includes any covariates that experts might consider relevant such as comorbidities and current or prior medication use.

Our analysis included 118 180 new users of duloxetine and 152 298 new users of sertraline, and produced a propensity score-adjusted hazard ratio of 1.13 (95% CI: 0.81–1.61). This result stands agreement with Lee *et al*. [18], who included 76 920 new users of the class containing duloxetine and 582 650 new users of the class containing sertraline, and reports an adjusted hazard ratio of 1.01 (95% CI: 0.90–1.12). Both studies also include sensitivity analyses that consider an alternative time-at-risk definition and show little variation in the estimate. We argue that the method used in both studies is of comparable rigour, and that our analysis meets the criteria for peer review, demonstrated by the publication of our studies using similar designs [19–21].

**Figure 3.**
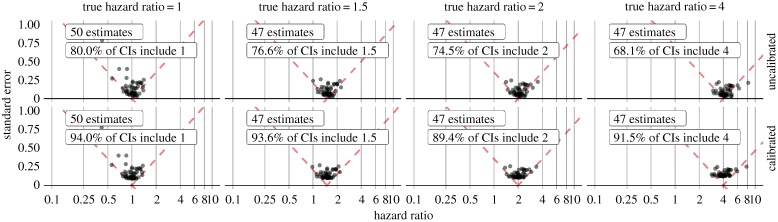
Evaluation of effect estimation between duloxetine and sertraline new users after stratification on the propensity scores before (top) and after (bottom) calibration. Each dot represents the hazard ratio and corresponding standard error for one of the negative (true hazard ratio = 1) or positive control (true hazard ratio greater than 1) outcomes. (Online version in colour.)

Figure 3 shows the estimates produced by applying the same analysis to a set of control outcomes (outcomes where the hazard ratio is known), while still comparing duloxetine to sertraline. This figure reveals the coverage of the uncalibrated 95% CI to be smaller than 95%. Calibrating the CIs using these observed operating characteristics restores near-nominal coverage.

Using the same calibration process, our hazard ratio for stroke becomes 1.11 (95% CI: 0.77–1.62), compared to the uncalibrated estimate of 1.13 (95% CI: 0.81–1.61). Although these estimates are similar, the empirical evaluation and calibration provide confidence that systematic error in our calibrated estimate remains small.

### (b) Results of all comparisons

With four databases, the potential number of effect size estimates is (4 × 5984 =) 23 936. We generate no risk estimate if at least one of the two treatment groups in a comparison contains fewer than 2500 persons, so the final count is 17 718 estimates. The full set of results are available in the electronic supplementary material and can be explored online at http://data.ohdsi.org/SystematicEvidence. The results of our evaluation and calibration using control outcomes, verified by cross-validation can be found in the electronic supplementary material. (The distribution of calibrated effect size estimates is also shown in figure 5*c*.)

### (c) Effect transitivity

If drug A has a statistically significant higher risk than drug B for a particular outcome, and drug B has a statistically significant higher risk than C for that same outcome, we expect A to have a statistically significant higher risk than C. In total, we identified 755 such A–B–C combinations, of which for 722 triplets (96%), the transitivity property held.

### (d) Between-database consistency

**Figure 4.**
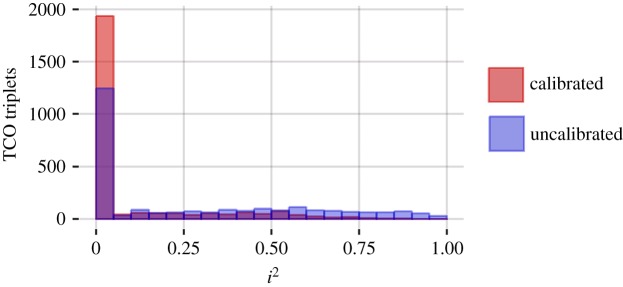
*I*^2^ distribution for all 2570 TCO triplets for which there was enough data in all four databases. Blue shows the distribution before calibration, red shows the distribution after calibration. (Online version in colour.)

Our previous work has suggested remarkably high heterogeneity when uncalibrated but identical observational study designs are implemented in different databases [22]. In the present context, ideally, calibrated effects estimated across the four observational databases would be relatively consistent. For the 2570 target–comparator–outcome (TCO) triplets having sufficient data in all four databases, we compute the *I*^2^ heterogeneity metric [23]. An *I*^2^ of zero means no between-database heterogeneity is observed. Across databases, 83% of calibrated estimates have an *I*^2^ below 0.25; see figure 4 for a complete histogram. By contrast, and in line with our previous work, only 58% of the estimates have an *I*^2^ below 0.25 when no calibration is applied.

### (e) Consistency with established knowledge

An additional test of validity compares our results with the current literature. Gartlehner *et al*. [24] systematically review comparative effects of antidepressant treatments based on RCTs and observational studies. Five findings emerge from the RCTs: (1) sertraline has higher risk of diarrhoea than comparators; (2) venlafaxine has higher risk of nausea than selective serotonin reuptake inhibitors (SSRIs); (3) there is no difference in nausea between duloxetine and paroxetine or fluoxetine; (4) paroxetine has higher rate of sexual dysfunction than fluoxetine and sertraline and (5) bupropion has lower incidence of sexual dysfunction than fluoxetine, paroxetine, and sertraline. Our result set correctly identified findings (1) through (4), as discussed in the electronic supplementary material—implying substantial but not perfect agreement with the literature. Electronic supplementary material, figure S3 also compares our results for finding (1) to estimates from RCTs reported in a systematic review on the topic [25], showing agreement as well as greater precision in our estimates due to the much larger sample size.

Finding (5) follows from five RCTs that demonstrated a significantly lower rate of sexual adverse events in patients exposed to bupropion relative to SSRIs. Clinical guidelines suggest bupropion as an alternative treatment if a patient experiences sexual side effects with an SSRI medication [26] and other supporting trials recommend bupropion for patients for whom sexual dysfunction is a concern [27]. From our result set, three databases return increased risks associated with bupropion relative to sertraline and fluoxetine. For example, in CCAE, the calibrated hazard ratio for decreased libido between bupropion and sertraline new users is 1.43 (1.09–1.89) and 1.42 (1.10–1.84) relative to fluoxetine new users. Channelling bias due to unmeasured baseline characteristics, such as sexual behaviour, may explain this discordant finding.

### (f) Comparing the distribution of estimates with the literature

**Figure 5.**
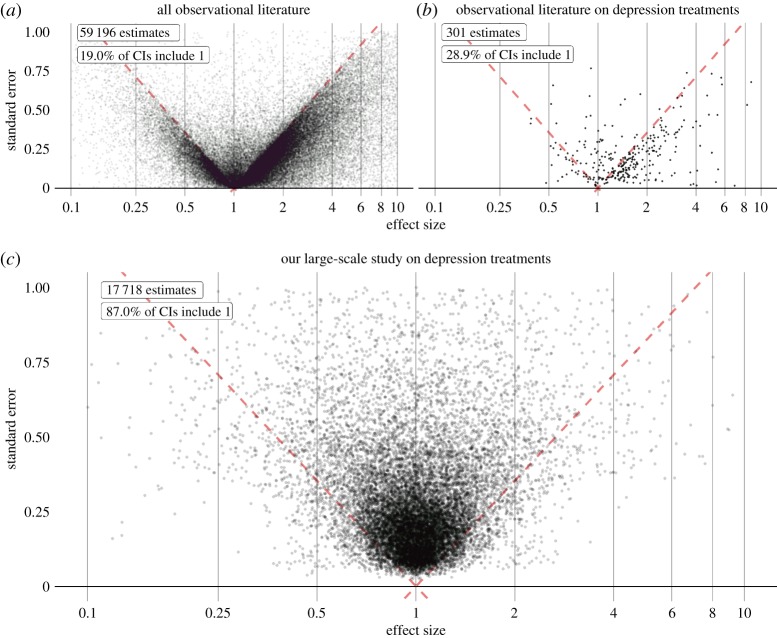
Effect size estimates from the literature (*a*, *b*) and the study described in this paper (*c*). Each dot represents a single estimate, such as relative risk, odds ratio or hazard ratio, and corresponding standard error (linearly related to the width of the asymptotic CI). Estimates below the red dashed line have a CI that excludes 1, suggesting a non-null effect. Plot (*a*) shows estimates extracted from the abstracts of all observational research papers in MEDLINE, plot (*b*) shows only the subset of those that are related to depression treatments. Plot (*c*) shows estimated and calibrated hazard ratios for comparisons between depression treatments for various health outcomes of interest, generated from observational data in a single study using a systematic process. An online interactive visualization enables readers to explore these results in detail, including individual study artefacts for the estimates we generated (http://data.ohdsi.org/SystematicEvidence). (Online version in colour.)

To compare our approach to the current scientific process, we show the distribution of effect size estimates from observational studies reported in the literature (figure 5*a*), the subset of estimates for depression treatments (figure 5*b*) and compare it against estimates produced in the high-throughput study described in this paper (figure 5*c*). At least three observations emerge from the current corpus of published observational studies. First, *the vast majority of effect estimates in literature abstracts (greater than 80%) have a CI that excludes 1* (i.e. statistically significant effects at *p* < 0.05). One explanation posits that researchers select hypotheses to test that have high *a priori* probabilities of being true. Another explanation is that observational studies are vulnerable to bias, for example due to confounding, selection bias and measurement error, that can easily lead to statistically significant but erroneous results [28]. Yet another explanation is that there is a tendency to only report results when the CI excludes 1, resulting in publication bias. This ties into our second observation: *in evidence reported in the literature, there is a sharp boundary between the regions where the CI does and does not include 1*, suggesting that publication bias is pervasive. Third, when focusing on one specific area of interest (figure 5*b*), in this case depression treatments, *the literature is sparse* compared to the more exhaustive approach taken in our study. Few of the questions that could have been asked are truly answered in the literature, perhaps because the current process is too inefficient, slow or because of the demonstrated publication bias.

## 4. Discussion

The distribution of estimates extracted from the literature (figure 5*a*,*b*) exposes several concerns: answers to many relevant questions are missing, either because they have not yet been investigated or because publication bias hides effect sizes close to or equal to one. In addition, evidence that is present is unreliable for two reasons. One reason is the evident publication bias, making a high false-positive rate likely [2]. In aggregate, published observational research is akin to data fishing at a large scale; by reporting primarily ‘statistically significant’ results and hiding others, spurious results due to random error appear legitimate because no adjustment is possible for the hidden multiple testing. The second reason is the proliferation of observational study bias [28]. Indeed, observational research literature stands replete with multiple studies reporting statistically significant results in conflicting directions, even when employing the same data [29–38].

Applying a high-throughput observational study design can address these problems, as we have demonstrated in our example study comparing the effects of depression treatments: first, the evidence from such a study can be produced and disseminated as a single unit, and thereby prevent publication bias from reducing the validity of our results. Second, the inclusion of control hypotheses allows for evaluation of our study, measuring its operating characteristics such as coverage of the CI. We even use these measures to calibrate our results to restore nominal characteristics, as confirmed in our experimental results. Consequently, our estimates have a markedly different distribution compared with those found in the current literature, as demonstrated by comparing figure 5*a–c*.

### (a) Does quantity come at the cost of quality?

A potential criticism to our approach is that addressing many research questions at once is at odds with thoughtful study design for any single question, and therefore likely leads to lower quality research. However, each of our analyses is of high quality, sufficient to pass peer review as demonstrated by comparing our duloxetine–sertraline–stroke example to a published study and our prior publications using similar designs. Similarly, our evaluation using control hypotheses provides further confidence in the quality of our designs and goes above and beyond the recent tentative calls to include negative controls in observational studies [39,40].

In fact, we believe unfettered freedom to customize a study for any research question is one of the main causes of the lack of reproducibility of observational study results, leading us to the situation portrayed in figure 5*a*. Our challenge to the scientific community is to point out changes to our study design that researchers believe to be necessary when answering a particular question. Such changes should be evaluated objectively on their merit, for example, using control hypotheses, and if proven to indeed improve quality, can be incorporated in a systematic way in the overall study design. Thus, science can move forward in a meaningful way, out of the current crisis.

### (b) Limitations

We require our negative controls to be truly negative, but we rarely have definitive evidence of the absence of a causal relationship. We must assume that a lack of evidence of an effect for well-studied treatments and outcomes implies evidence of a lack of effect. In reality, some of our negative controls could prove to be positive at a future point in time.

In our evaluation and calibration procedure, we require that the controls and the hypotheses of interest are exchangeable in certain aspects. We address this by choosing controls with the same target and comparator definitions, only differing in the outcome. However, negative controls could exhibit different bias than the outcomes of interest. Note that we do not assume the biases for these controls exactly equal the biases for the outcomes of interest, rather we assume only that biases draw from the same distribution. Unfortunately, we do not know for certain that this broad assumption holds. Furthermore, our positive controls fail to reflect bias due to unmeasured confounding other than that present for the negative controls on which they were based. However, we argue that detection of bias that may not fully represent all bias is better than ignoring bias completely.

A further limitation of observational research in general is that evidence can be generated only for those treatments and outcomes that are captured during interactions with the healthcare system and are reflected in the data. Some outcomes cannot be studied using these data. For example, we could not study reduction in depression symptoms as a possible outcome of treatment. Unmeasured confounding factors, as may have biased the estimate of the effect of bupropion on sexual dysfunction, remain a potential threat to the reliability of observational studies.

### (c) How to use our results

Despite the limitations of observational data, they represent a critical component in improving the healthcare evidence base. Even though depression treatments have been extensively studied with hundreds of clinical trials, there is still much we do not know about the comparative effectiveness (including safety and tolerability) of alternative treatments. Evidence from our observational study can provide a reference to compare what we have learned in trials with what is observed in the real world. The evidence can also be a primary source when trials are unavailable, underpowered or non-generalizable to the target population of interest. Ideally, our results can contribute to a more complete evaluation of the balance of risks and benefits of treatments, although as noted earlier, the data often do not inform sufficiently on benefits.

We believe that our results should be used similarly to how one would use results currently scattered across observational research papers in the literature, which is typically a hypothesis-driven process. We purposely do *not* correct for multiple hypotheses in our results because that can only be done once a hypothesis or set of hypotheses is chosen. As when using results from the literature, it is important to consider false positives when faced with multiple testing, and our results readily allow for adjustment for multiple testing, because we have disseminated all results. Note that such adjustments are not possible when using evidence scattered in the literature, because many studies that should have been considered were never published due to publication bias. If readers dredge our results set looking for the most statistically significant ones, appropriate interpretation will require a multiplicity correction (e.g. Bonferroni or false discovery rate analysis). We believe that the value of our results, however, lies not in finding a few very significant ones, but in having available results that are poised to answer specific questions with as little bias as currently possible in observational research.

### (d) Improving our study

While our choice of methods to address confounding (data-driven propensity score matching) and residual bias (CI calibration) can be considered the current best practice, future studies could replace these with improved methods. One possible direction could be the use of self-controlled designs such as the self-controlled case series [41]. Other possible future additions include estimation of absolute risk (instead of relative risk) to better facilitate benefit–risk assessment, as well as evaluating effect heterogeneity across subpopulations. In fact, we sincerely hope that observational researchers will move their focus from performing one-off studies to refining the high-throughput approach as described in this paper. Rather than each researcher working in isolation, we hope the scientific community will come together to build the process that generates evidence. To facilitate this, we have made all software necessary to execute this study available as open source (https://github.com/OHDSI/StudyProtocols/tree/master/LargeScalePopEst).

## 5. Conclusion

We propose a paradigm shift in how researchers generate and disseminate evidence from observational data. The current paradigm centres on generating one estimate at a time using a unique study design with unknown operating characteristics and publishing estimates one at a time through a dissemination process with clear limitations. The new paradigm advocates for larger-scale studies that produce concurrent results for multiple hypotheses using consistent and standardized methods, allowing evaluation, calibration and unbiased dissemination to generate a more reliable and complete evidence base than was previously possible. The results are poised for answering specific questions, able to be adjusted for multiple hypotheses as appropriate to the question at hand. Clinicians, regulators and other medical decision makers can improve the care for patients by making well-informed decisions based on this evidence, and every treatment a patient receives becomes the basis for further evidence.

## Supplementary Material

Supplementary materials

## Supplementary Material

Data file S1

## Supplementary Material

Data file S2

## Data accessibility

The full set of results are available in the electronic supplementary material and can be explored online at http://data.ohdsi.org/SystematicEvidence.
