# Supplementary material for: Improving reproducibility by using high-throughput observational studies with empirical calibration
Source: Philos Trans A Math Phys Eng Sci. 2018 Aug 6;376(2128):20170356. doi: 10.1098/rsta.2017.0356 (PMC6107542; doi:10.1098/rsta.2017.0356)
Supplement: Supplementary materials [file rsta20170356supp1.docx]

**Supplementary materials**

**Table of contents**

[Document S1. Exposure definitions 2](#_Toc507400638)

[Document S2. Outcome definitions 7](#_Toc507400639)

[Acute liver injury 7](#_Toc507400640)

[Acute myocardial infarction 8](#_Toc507400641)

[Alopecia 8](#_Toc507400642)

[Constipation 9](#_Toc507400643)

[Decreased libido 9](#_Toc507400644)

[Delirium 10](#_Toc507400645)

[Diarrhea 10](#_Toc507400646)

[Fracture 11](#_Toc507400647)

[Gastrointestinal hemhorrage 11](#_Toc507400648)

[Hyperprolactinemia 12](#_Toc507400649)

[Hyponatremia 12](#_Toc507400650)

[Hypotension 13](#_Toc507400651)

[Hypothyroidism 14](#_Toc507400652)

[Insomnia 14](#_Toc507400653)

[Nausea 15](#_Toc507400654)

[Open-angle glaucoma 15](#_Toc507400655)

[Seizure 16](#_Toc507400656)

[Stroke 16](#_Toc507400657)

[Suicide and suicidal ideation 17](#_Toc507400658)

[Tinnitus 18](#_Toc507400659)

[Ventricular arrhythmia and sudden cardiac death 18](#_Toc507400660)

[Vertigo 19](#_Toc507400661)

[Document S3. Consistency with gold standard from randomized controlled trials 20](#_Toc507400662)

[Figure S1. Estimates for all control hypotheses before and after calibration 22](#_Toc507400663)

[Figure S2. Leave-one-out cross-validation of calibration 23](#_Toc507400664)

[Figure S3. Comparison of results from RCTs and our observational study 24](#_Toc507400665)

[References 25](#_Toc507400666)

# Document S1. Exposure definitions

The target and comparator groups consisted of new users of the treatments listed in Table 1, which are identified using the codes specified in tables S1.1-S1.4. For both cohorts we restrict to people with a prior diagnosis of depression, and no prior history of bipolar disorder or schizophrenia.

| **Concept ID** | **Concept Name** |
| --- | --- |
| 4152280 | Major depressive disorder |
| 435783 | Schizophrenia |
| 436665 | Bipolar disorder |

**Table S1.1.** Concepts used to identify prior history of depression, schizophrenia, and bipolar disorders. All descendants of these concepts are also considered.

| **Concept ID** | **RxNorm ID** | **Concept Name** |
| --- | --- | --- |
| 710062 | 704 | Amitriptyline |
| 750982 | 42347 | Bupropion |
| 797617 | 2556 | Citalopram |
| 717607 | 734064 | Desvenlafaxine |
| 738156 | 3638 | Doxepin |
| 715259 | 72625 | duloxetine |
| 715939 | 321988 | Escitalopram |
| 755695 | 4493 | Fluoxetine |
| 725131 | 15996 | Mirtazapine |
| 721724 | 7531 | Nortriptyline |
| 722031 | 32937 | Paroxetine |
| 739138 | 36437 | Sertraline |
| 703547 | 10737 | Trazodone |
| 743670 | 39786 | venlafaxine |
| 40234834 | 1086769 | vilazodone |

**Table S1.2.** The 15 included drugs and their concept identifiers. All descendants of these concepts are also considered.

| **Concept ID** | **Concept Name** |
| --- | --- |
| 4119335 | Analytical psychology |
| 4084202 | Anti-criminal psychotherapy |
| 4079608 | Anti-suicide psychotherapy |
| 4048385 | Brief group psychotherapy |
| 4295027 | Brief solution focused psychotherapy |
| 4299728 | Client-centered psychotherapy |
| 4164790 | Conjoint psychotherapy |
| 4208314 | Couple psychotherapy |
| 4083706 | Crisis intervention |
| 4083131 | Daily life psychotherapy |
| 4121662 | Developmental psychodynamic psychotherapy |
| 4226276 | Eclectic psychotherapy |
| 4258834 | Educational psychotherapy |
| 4148765 | Encounter group therapy |
| 2007747 | Exploratory verbal psychotherapy |
| 4137086 | Expressed emotion family therapy |
| 4048387 | Expressive psychotherapy |
| 4173581 | Extended family therapy |
| 46286403 | Family intervention for psychosis |
| 2213546 | Family psychotherapy (conjoint psychotherapy) (with patient present) |
| 4028920 | Family psychotherapy procedure |
| 46286330 | Focal psychodynamic therapy |
| 4226275 | Formal psychological therapy |
| 45765516 | Functional family therapy |
| 4079939 | Functional psychotherapy |
| 4079500 | General psychotherapy |
| 4117915 | Generic Jungian-based therapy |
| 4100341 | Group analytical psychotherapy |
| 44808677 | Group cognitive behavioural therapy |
| 4136352 | Group marathon therapy |
| 4268909 | Group primal therapy |
| 4296166 | Group psychotherapy |
| 2213548 | Group psychotherapy (other than of a multiple-family group) |
| 2617477 | Group psychotherapy other than of a multiple-family group, in a partial hospitalization setting, approximately 45 to 50 minutes |
| 4196062 | Group reassurance |
| 2213554 | Individual psychophysiological therapy incorporating biofeedback training by any modality (face-to-face with the patient), with psychotherapy (eg, insight oriented, behavior modifying or supportive psychotherapy); 30 minutes |
| 2213555 | Individual psychophysiological therapy incorporating biofeedback training by any modality (face-to-face with the patient), with psychotherapy (eg, insight oriented, behavior modifying or supportive psychotherapy); 45 minutes |
| 4088889 | Individual psychotherapy |
| 2007730 | Individual psychotherapy |
| 4103512 | Interactive group medical psychotherapy |
| 2617478 | Interactive group psychotherapy, in a partial hospitalization setting, approximately 45 to 50 minutes |
| 4221997 | Interactive individual medical psychotherapy |
| 40482841 | Interpersonal psychotherapy |
| 4119334 | Jungian-based therapy |
| 4118797 | Long-term exploratory psychotherapy |
| 4118798 | Long-term psychodynamic psychotherapy |
| 44792695 | Marital psychotherapy |
| 2213547 | Multiple-family group psychotherapy |
| 4118800 | Narrative family psychotherapy |
| 4242119 | Occupational social therapy |
| 2007749 | Other individual psychotherapy |
| 2007750 | Other psychotherapy and counselling |
| 45887728 | Other Psychotherapy Procedures |
| 45763911 | Parent-infant psychotherapy |
| 2007746 | Play psychotherapy |
| 4083133 | Potential suicide care |
| 4084195 | Provocative therapy |
| 2213544 | Psychoanalysis |
| 2007731 | Psychoanalysis |
| 4114491 | Psychoanalytic and psychodynamic therapy |
| 4202234 | Psychodrama |
| 2007763 | Psychodrama |
| 4199042 | Psychodynamic psychotherapy |
| 4128268 | Psychodynamic-interpersonal psychotherapy |
| 4118801 | Psychotherapeutic approaches using specific settings |
| 4327941 | Psychotherapy |
| 4083129 | Psychotherapy - behavioral |
| 4079938 | Psychotherapy - cognitive |
| 45889353 | Psychotherapy for crisis |
| 45888237 | Psychotherapy for Crisis Services and Procedures |
| 43527991 | Psychotherapy for crisis; each additional 30 minutes (List separately in addition to code for primary service) |
| 43527990 | Psychotherapy for crisis; first 60 minutes |
| 45887951 | Psychotherapy Services and Procedures |
| 2108571 | Psychotherapy services provided (MDD, MDD ADOL) |
| 43527986 | Psychotherapy, 30 minutes with patient and/or family member |
| 43527987 | Psychotherapy, 30 minutes with patient and/or family member when performed with an evaluation and management service (List separately in addition to the code for primary procedure) |
| 43527904 | Psychotherapy, 45 minutes with patient and/or family member |
| 43527988 | Psychotherapy, 45 minutes with patient and/or family member when performed with an evaluation and management service (List separately in addition to the code for primary procedure) |
| 43527905 | Psychotherapy, 60 minutes with patient and/or family member |
| 43527989 | Psychotherapy, 60 minutes with patient and/or family member when performed with an evaluation and management service (List separately in addition to the code for primary procedure) |
| 4148398 | Psychotherapy/sociotherapy |
| 4083130 | Rehabilitation for disabling psychiatric problem |
| 44791916 | Relationship psychosexual therapy |
| 4265313 | Relationship psychotherapy |
| 4084201 | Samaritans advisory service |
| 4233181 | Sensate focus technique |
| 4272803 | Sexual psychotherapy |
| 4035812 | Sexual psychotherapy, female therapist - female patient |
| 4012488 | Sexual psychotherapy, female therapist - male patient |
| 4132436 | Sexual psychotherapy, group |
| 4143316 | Sexual psychotherapy, group, all female |
| 4219683 | Sexual psychotherapy, group, all male |
| 4151904 | Sexual psychotherapy, group, male and female |
| 4278094 | Sexual psychotherapy, male therapist - female patient |
| 4249602 | Sexual psychotherapy, male therapist - male patient |
| 4234476 | Sexual surrogate therapy |
| 4179241 | Short-term psychodynamic therapy |
| 4234402 | Social psychotherapy |
| 4128406 | Specific task orientated psychotherapy |
| 4080044 | Stimulative psychotherapy |
| 4262582 | Structural family psychotherapy |
| 4263758 | Structural psychotherapy |
| 4126653 | Supportive expressive psychodynamic psychotherapy |
| 4311943 | Supportive verbal psychotherapy |
| 2007748 | Supportive verbal psychotherapy |
| 4225728 | Suppressive psychotherapy |
| 4080048 | Therapeutic psychology |
| 44808259 | Therapeutic role play |

**Table S1.3.** Concepts used to identify psychotherapy.

| **Concept ID** | **Concept Name** |
| --- | --- |
| 4111663 | Bilateral electroconvulsive therapy |
| 4030840 | Electroconvulsive therapy |
| 2108578 | Electroconvulsive therapy (ECT) provided (MDD) |
| 2213552 | Electroconvulsive therapy (includes necessary monitoring) |
| 4020981 | Electronarcosis |
| 4210144 | First treatment in a course of electroconvulsive therapy |
| 4336318 | Multiple electroconvulsive therapy |
| 4332436 | Multiple monitored electroconvulsive therapy |
| 2007728 | Other electroshock therapy |
| 44508134 | Other specified electroconvulsive therapy |
| 2108579 | Patient referral for electroconvulsive therapy (ECT) documented (MDD) |
| 2007727 | Subconvulsive electroshock therapy |
| 4004830 | Subconvulsive electroshock therapy |
| 4210145 | Subsequent treatment in a course of electroconvulsive therapy |

**Table S1.4.** Concepts used to identify electroconvulsive therapy.

# Document S2. Outcome definitions

This section describes the algorithms we use to identify occurrences of the 22 outcomes of interest. The algorithms are framed in the context of the OMOP Common Data Model[1] (CDM) version 5. The CDM uses a standardized terminology for encoding all information. For more information on the CDM and the standard vocabulary see <http://ohdsi.org>. The computer-executable version of these algorithms is part of the study R package: <https://github.com/OHDSI/StudyProtocols/tree/master/LargeScalePopEst>

## Acute liver injury

Note: This algorithm uses the set of codes identified by Udo et al. [2]

Initial Event Cohort

People having any of the following: 

- a condition occurrence of acute liver injury^1^
  - for the first time in the person's history
  - visit occurrence is any of: Emergency Room Visit, Inpatient Visit

with continuous observation of at least 0 days prior and 0 days after event index date, and limit initial events to: **earliest event per person.**

For people matching the Primary Events, include:

Having all of the following criteria:

- exactly 0 occurrences of a condition occurrence of acute liver injury exclusion concepts^2^

starting between 365 days Before and 60 days After event index date

Limit cohort of initial events to: **earliest event per person.**

Limit qualifying cohort to: **earliest event per person.**

No end date strategy selected. By default, the cohort end date will be the end of the observation period that contains the index event.

Appendix 1: Concept Set Definitions

1. acute liver injury

| **Concept Id** | **Concept Name** | **Domain** | **Vocabulary** | **Excluded** | **Descendants** | **Mapped** |
| --- | --- | --- | --- | --- | --- | --- |
| 200763 | Chronic hepatitis | Condition | SNOMED | YES | YES | NO |
| 377604 | Hepatic coma | Condition | SNOMED | NO | YES | NO |
| 196029 | Hepatic coma due to viral hepatitis | Condition | SNOMED | YES | YES | NO |
| 4337543 | Hepatic necrosis | Condition | SNOMED | NO | YES | NO |
| 194087 | Hepatitis due to infection | Condition | SNOMED | YES | YES | NO |
| 196455 | Hepatorenal syndrome | Condition | SNOMED | NO | YES | NO |
| 194990 | Inflammatory disease of liver | Condition | SNOMED | NO | YES | NO |
| 4291005 | Viral hepatitis | Condition | SNOMED | YES | YES | NO |

2. acute liver injury exclusion concepts

| **Concept Id** | **Concept Name** | **Domain** | **Vocabulary** | **Excluded** | **Descendants** | **Mapped** |
| --- | --- | --- | --- | --- | --- | --- |
| 192956 | Cholecystitis | Condition | SNOMED | NO | YES | NO |
| 200763 | Chronic hepatitis | Condition | SNOMED | NO | YES | NO |
| 4212540 | Chronic liver disease | Condition | SNOMED | NO | YES | NO |
| 197917 | Disorder of biliary tract | Condition | SNOMED | NO | YES | NO |
| 192353 | Disorder of gallbladder | Condition | SNOMED | NO | YES | NO |
| 192963 | Disorder of pancreas | Condition | SNOMED | NO | YES | NO |
| 196456 | Gallstone | Condition | SNOMED | NO | YES | NO |
| 4130518 | Neoplasm of liver | Condition | SNOMED | NO | YES | NO |
| 4291005 | Viral hepatitis | Condition | SNOMED | NO | YES | NO |

## Acute **myocardial** infarction

Initial Event Cohort

People having any of the following: 

- a condition occurrence of Acute MI^1^
  - for the first time in the person's history
  - condition type is any of: Inpatient detail - primary, Inpatient header - primary, Primary Condition, Inpatient detail - 1st position, Inpatient header - 1st position
  - visit occurrence is any of: Emergency Room Visit, Inpatient Visit

with continuous observation of at least 0 days prior and 0 days after event index date, and limit initial events to: **earliest event per person.**

Limit qualifying cohort to: **earliest event per person.**

No end date strategy selected. By default, the cohort end date will be the end of the observation period that contains the index event.

Appendix 1: Concept Set Definitions

1. Acute MI

| **Concept Id** | **Concept Name** | **Domain** | **Vocabulary** | **Excluded** | **Descendants** | **Mapped** |
| --- | --- | --- | --- | --- | --- | --- |
| 4329847 | Myocardial infarction | Condition | SNOMED | NO | YES | NO |
| 314666 | Old myocardial infarction | Condition | SNOMED | YES | YES | NO |

## Alopecia

Initial Event Cohort

People having any of the following: 

- a condition occurrence of Alopecia^1^
  - for the first time in the person's history

with continuous observation of at least 0 days prior and 0 days after event index date, and limit initial events to: **earliest event per person.**

Limit qualifying cohort to: **earliest event per person.**

No end date strategy selected. By default, the cohort end date will be the end of the observation period that contains the index event.

Appendix 1: Concept Set Definitions

1. Alopecia

| **Concept Id** | **Concept Name** | **Domain** | **Vocabulary** | **Excluded** | **Descendants** | **Mapped** |
| --- | --- | --- | --- | --- | --- | --- |
| 133280 | Alopecia | Condition | SNOMED | NO | YES | NO |
| 133959 | Syphilitic alopecia | Condition | SNOMED | YES | YES | NO |

## Constipation

Note: This algorithm requires the occurrence of 2 or more diagnoses, as recommended by Mody et al. [3]

Initial Event Cohort

People having any of the following: 

- a condition occurrence of Constipation^1^
  - for the first time in the person's history

with continuous observation of at least 0 days prior and 0 days after event index date, and limit initial events to: **earliest event per person.**

Limit qualifying cohort to: **earliest event per person.**

No end date strategy selected. By default, the cohort end date will be the end of the observation period that contains the index event.

Appendix 1: Concept Set Definitions

1. Constipation

| **Concept Id** | **Concept Name** | **Domain** | **Vocabulary** | **Excluded** | **Descendants** | **Mapped** |
| --- | --- | --- | --- | --- | --- | --- |
| 75860 | Constipation | Condition | SNOMED | NO | YES | NO |

## Decreased libido

Initial Event Cohort

People having any of the following: 

- a condition occurrence of Decreased libido^1^
  - for the first time in the person's history

with continuous observation of at least 0 days prior and 0 days after event index date, and limit initial events to: **earliest event per person.**

Limit qualifying cohort to: **earliest event per person.**

No end date strategy selected. By default, the cohort end date will be the end of the observation period that contains the index event.

Appendix 1: Concept Set Definitions

1. Decreased libido

| **Concept Id** | **Concept Name** | **Domain** | **Vocabulary** | **Excluded** | **Descendants** | **Mapped** |
| --- | --- | --- | --- | --- | --- | --- |
| 436246 | Reduced libido | Condition | SNOMED | NO | YES | NO |

## Delirium

Note: This algorithm relies on diagnosis codes associated with hospitalization. This approach may lead to underreporting, as described by McCoy et al. [4]

Initial Event Cohort

People having any of the following: 

- a condition occurrence of Delirium^1^
  - for the first time in the person's history
  - visit occurrence is any of: Emergency Room Visit, Inpatient Visit

with continuous observation of at least 0 days prior and 0 days after event index date, and limit initial events to: **earliest event per person.**

Limit qualifying cohort to: **earliest event per person.**

No end date strategy selected. By default, the cohort end date will be the end of the observation period that contains the index event.

Appendix 1: Concept Set Definitions

1. Delirium

| **Concept Id** | **Concept Name** | **Domain** | **Vocabulary** | **Excluded** | **Descendants** | **Mapped** |
| --- | --- | --- | --- | --- | --- | --- |
| 377830 | Alcohol withdrawal delirium | Condition | SNOMED | YES | YES | NO |
| 373995 | Delirium | Condition | SNOMED | NO | YES | NO |

## Diarrhea

Note: This algorithm follows Broder et al. [5]

Initial Event Cohort

People having any of the following: 

- a condition occurrence of Diarrhea^1^
  - for the first time in the person's history

with continuous observation of at least 0 days prior and 0 days after event index date, and limit initial events to: **earliest event per person.**

Limit qualifying cohort to: **earliest event per person.**

No end date strategy selected. By default, the cohort end date will be the end of the observation period that contains the index event.

Appendix 1: Concept Set Definitions

1. Diarrhea

| **Concept Id** | **Concept Name** | **Domain** | **Vocabulary** | **Excluded** | **Descendants** | **Mapped** |
| --- | --- | --- | --- | --- | --- | --- |
| 196523 | Diarrhea | Condition | SNOMED | NO | YES | NO |
| 80141 | Functional diarrhea | Condition | SNOMED | NO | YES | NO |

## Fracture

Note: This algorithm follows Lanteigne et al. [6]

Initial Event Cohort

People having any of the following: 

- a condition occurrence of Fracture^1^
  - for the first time in the person's history

with continuous observation of at least 0 days prior and 0 days after event index date, and limit initial events to: **earliest event per person.**

Limit qualifying cohort to: **earliest event per person.**

No end date strategy selected. By default, the cohort end date will be the end of the observation period that contains the index event.

Appendix 1: Concept Set Definitions

1. Fracture

| **Concept Id** | **Concept Name** | **Domain** | **Vocabulary** | **Excluded** | **Descendants** | **Mapped** |
| --- | --- | --- | --- | --- | --- | --- |
| 435093 | Closed fracture of femur | Condition | SNOMED | NO | YES | NO |
| 441974 | Closed fracture of forearm | Condition | SNOMED | NO | YES | NO |
| 4230399 | Closed fracture of hip | Condition | SNOMED | NO | YES | NO |
| 441422 | Closed fracture of humerus | Condition | SNOMED | NO | YES | NO |
| 439166 | Closed fracture of radius | Condition | SNOMED | NO | YES | NO |
| 4278672 | Fracture of forearm | Condition | SNOMED | NO | YES | NO |
| 442619 | Fracture of humerus | Condition | SNOMED | NO | YES | NO |
| 433856 | Fracture of neck of femur | Condition | SNOMED | NO | YES | NO |
| 4131595 | Fracture of radius | Condition | SNOMED | NO | YES | NO |
| 73571 | Pathological fracture | Condition | SNOMED | NO | YES | NO |

## Gastrointestinal hemhorrage

Initial Event Cohort

People having any of the following: 

- a condition occurrence of Gastrointestinal hemorrhage^1^
  - for the first time in the person's history
  - condition type is any of: Inpatient detail - primary, Inpatient header - primary, Primary Condition, Inpatient detail - 1st position, Inpatient header - 1st position
  - visit occurrence is any of: Emergency Room Visit, Inpatient Visit

with continuous observation of at least 0 days prior and 0 days after event index date, and limit initial events to: **earliest event per person.**

Limit qualifying cohort to: **earliest event per person.**

No end date strategy selected. By default, the cohort end date will be the end of the observation period that contains the index event.

Appendix 1: Concept Set Definitions

1. Gastrointestinal hemorrhage

| **Concept Id** | **Concept Name** | **Domain** | **Vocabulary** | **Excluded** | **Descendants** | **Mapped** |
| --- | --- | --- | --- | --- | --- | --- |
| 4280942 | Acute gastrojejunal ulcer with perforation | Condition | SNOMED | NO | YES | NO |
| 28779 | Bleeding esophageal varices | Condition | SNOMED | NO | YES | NO |
| 198798 | Dieulafoy's vascular malformation | Condition | SNOMED | NO | YES | NO |
| 4112183 | Esophageal varices with bleeding, associated with another disorder | Condition | SNOMED | NO | YES | NO |
| 194382 | External hemorrhoids | Condition | SNOMED | NO | NO | NO |
| 192671 | Gastrointestinal hemorrhage | Condition | SNOMED | NO | YES | NO |
| 196436 | Internal hemorrhoids | Condition | SNOMED | NO | NO | NO |
| 4338225 | Peptic ulcer with perforation | Condition | SNOMED | NO | YES | NO |
| 194158 | Perinatal gastrointestinal hemorrhage | Condition | SNOMED | YES | YES | NO |

## Hyperprolactinemia

Initial Event Cohort

People having any of the following: 

- a condition occurrence of Hyperprolactinemia^1^
  - for the first time in the person's history

with continuous observation of at least 0 days prior and 0 days after event index date, and limit initial events to: **earliest event per person.**

Limit qualifying cohort to: **earliest event per person.**

No end date strategy selected. By default, the cohort end date will be the end of the observation period that contains the index event.

Appendix 1: Concept Set Definitions

1. Hyperprolactinemia

| **Concept Id** | **Concept Name** | **Domain** | **Vocabulary** | **Excluded** | **Descendants** | **Mapped** |
| --- | --- | --- | --- | --- | --- | --- |
| 4030186 | Hyperprolactinemia | Condition | SNOMED | NO | YES | NO |

## Hyponatremia

Note: The algorithm here relies on the recording of diagnoses codes, and might not have high sensitivity as remarked by Shea et al.

Initial Event Cohort

People having any of the following: 

- a condition occurrence of Hyponatremia^1^
  - for the first time in the person's history
- a measurement of Serum sodium^2^
  - for the first time in the person's history
  - with value as number < 136
  - unit is any of: millimole per liter

with continuous observation of at least 0 days prior and 0 days after event index date, and limit initial events to: **earliest event per person.**

Limit qualifying cohort to: **earliest event per person.**

No end date strategy selected. By default, the cohort end date will be the end of the observation period that contains the index event.

Appendix 1: Concept Set Definitions

1. Hyponatremia

| **Concept Id** | **Concept Name** | **Domain** | **Vocabulary** | **Excluded** | **Descendants** | **Mapped** |
| --- | --- | --- | --- | --- | --- | --- |
| 435515 | Hypo-osmolality and or hyponatremia | Condition | SNOMED | NO | YES | NO |

2. Serum sodium

| **Concept Id** | **Concept Name** | **Domain** | **Vocabulary** | **Excluded** | **Descendants** | **Mapped** |
| --- | --- | --- | --- | --- | --- | --- |
| 3032987 | Sodium [Moles/volume] corrected for glucose in Serum or Plasma | Measurement | LOINC | NO | YES | NO |
| 46235784 | Sodium [Moles/volume] in Serum, Plasma or Blood | Measurement | LOINC | NO | YES | NO |
| 3019550 | Sodium serum/plasma | Measurement | LOINC | NO | YES | NO |

## Hypotension

Note: This algorithm follows Wernli et al. [7]

Initial Event Cohort

People having any of the following: 

- a condition occurrence of Hypotension^1^
  - for the first time in the person's history

with continuous observation of at least 0 days prior and 0 days after event index date, and limit initial events to: **earliest event per person.**

Limit qualifying cohort to: **earliest event per person.**

No end date strategy selected. By default, the cohort end date will be the end of the observation period that contains the index event.

Appendix 1: Concept Set Definitions

1. Hypotension

| **Concept Id** | **Concept Name** | **Domain** | **Vocabulary** | **Excluded** | **Descendants** | **Mapped** |
| --- | --- | --- | --- | --- | --- | --- |
| 4120275 | Drug-induced hypotension | Condition | SNOMED | NO | YES | NO |
| 317002 | Low blood pressure | Condition | SNOMED | NO | YES | NO |
| 314432 | Maternal hypotension syndrome | Condition | SNOMED | YES | YES | NO |
| 319041 | Orthostatic hypotension | Condition | SNOMED | NO | YES | NO |

## Hypothyroidism

Note: This algorithm requires the occurrences of 2 more diagnose codes, as recommended by Lu et al. [8]

Initial Event Cohort

People having any of the following: 

- a condition occurrence of Hypothyroidism^1^

with continuous observation of at least 0 days prior and 0 days after event index date, and limit initial events to: **all events per person.**

For people matching the Primary Events, include:

Having all of the following criteria:

- at least 2 occurrences of a condition occurrence of Hypothyroidism^1^

starting between 0 days Before and 90 days After event index date

Limit cohort of initial events to: **earliest event per person.**

Limit qualifying cohort to: **earliest event per person.**

No end date strategy selected. By default, the cohort end date will be the end of the observation period that contains the index event.

Appendix 1: Concept Set Definitions

1. Hypothyroidism

| **Concept Id** | **Concept Name** | **Domain** | **Vocabulary** | **Excluded** | **Descendants** | **Mapped** |
| --- | --- | --- | --- | --- | --- | --- |
| 140673 | Hypothyroidism | Condition | SNOMED | NO | YES | NO |

## Insomnia

Initial Event Cohort

People having any of the following: 

- a condition occurrence of Insomnia^1^
  - for the first time in the person's history

with continuous observation of at least 0 days prior and 0 days after event index date, and limit initial events to: **earliest event per person.**

Limit qualifying cohort to: **earliest event per person.**

No end date strategy selected. By default, the cohort end date will be the end of the observation period that contains the index event.

Appendix 1: Concept Set Definitions

1. Insomnia

| **Concept Id** | **Concept Name** | **Domain** | **Vocabulary** | **Excluded** | **Descendants** | **Mapped** |
| --- | --- | --- | --- | --- | --- | --- |
| 439708 | Disorders of initiating and maintaining sleep | Condition | SNOMED | NO | YES | NO |
| 436962 | Insomnia | Condition | SNOMED | NO | YES | NO |
| 4305303 | Sleep deprivation | Condition | SNOMED | NO | YES | NO |

## Nausea

Initial Event Cohort

People having any of the following: 

- a condition occurrence of Nausea^1^
  - for the first time in the person's history

with continuous observation of at least 0 days prior and 0 days after event index date, and limit initial events to: **earliest event per person.**

Limit qualifying cohort to: **earliest event per person.**

No end date strategy selected. By default, the cohort end date will be the end of the observation period that contains the index event.

Appendix 1: Concept Set Definitions

1. Nausea

| **Concept Id** | **Concept Name** | **Domain** | **Vocabulary** | **Excluded** | **Descendants** | **Mapped** |
| --- | --- | --- | --- | --- | --- | --- |
| 30284 | Motion sickness | Condition | SNOMED | YES | YES | NO |
| 31967 | Nausea | Condition | SNOMED | NO | YES | NO |

## Open-angle glaucoma

Note: This algorithm follows Stein et al. [9]

Initial Event Cohort

People having any of the following: 

- a condition occurrence of Open-angle glaucoma^1^
  - for the first time in the person's history

with continuous observation of at least 365 days prior and 0 days after event index date, and limit initial events to: **earliest event per person.**

For people matching the Primary Events, include:

Having all of the following criteria:

- at least 1 occurrences of a condition occurrence of Open-angle glaucoma^1^
  - provider specialty is any of: Ophthalmology, Optometry, Optician

starting between 1 days After and 365 days After event index date

Limit cohort of initial events to: **earliest event per person.**

Limit qualifying cohort to: **all events per person.**

No end date strategy selected. By default, the cohort end date will be the end of the observation period that contains the index event.

Appendix 1: Concept Set Definitions

1. Open-angle glaucoma

| **Concept Id** | **Concept Name** | **Domain** | **Vocabulary** | **Excluded** | **Descendants** | **Mapped** |
| --- | --- | --- | --- | --- | --- | --- |
| 432908 | Glaucomatocyclitic crisis | Condition | SNOMED | YES | YES | NO |
| 441561 | Low tension glaucoma | Condition | SNOMED | NO | YES | NO |
| 4216823 | Open angle with borderline findings | Condition | SNOMED | YES | YES | NO |
| 441284 | Open-angle glaucoma | Condition | SNOMED | NO | YES | NO |
| 4072218 | Secondary open-angle glaucoma | Condition | SNOMED | YES | YES | NO |

## Seizure

Note: This algorithm requires either inpatient or emergency room visits as recommended by Wu et al. [10]

Initial Event Cohort

People having any of the following: 

- a condition occurrence of Seizure and seizure disorder^1^
  - for the first time in the person's history
  - visit occurrence is any of: Emergency Room Visit, Inpatient Visit

with continuous observation of at least 0 days prior and 0 days after event index date, and limit initial events to: **earliest event per person.**

Limit qualifying cohort to: **earliest event per person.**

No end date strategy selected. By default, the cohort end date will be the end of the observation period that contains the index event.

Appendix 1: Concept Set Definitions

1. Seizure and seizure disorder

| **Concept Id** | **Concept Name** | **Domain** | **Vocabulary** | **Excluded** | **Descendants** | **Mapped** |
| --- | --- | --- | --- | --- | --- | --- |
| 380533 | Convulsions in the newborn | Condition | SNOMED | YES | YES | NO |
| 45757050 | Epilepsy in mother complicating pregnancy | Condition | SNOMED | YES | YES | NO |
| 377091 | Seizure | Condition | SNOMED | NO | YES | NO |
| 4029498 | Seizure disorder | Condition | SNOMED | NO | YES | NO |

## Stroke

Initial Event Cohort

People having any of the following: 

- a condition occurrence of Ischemic stroke^1^
  - for the first time in the person's history
  - visit occurrence is any of: Inpatient Visit

with continuous observation of at least 0 days prior and 0 days after event index date, and limit initial events to: **earliest event per person.**

Limit qualifying cohort to: **earliest event per person.**

No end date strategy selected. By default, the cohort end date will be the end of the observation period that contains the index event.

Appendix 1: Concept Set Definitions

1. Ischemic stroke

| **Concept Id** | **Concept Name** | **Domain** | **Vocabulary** | **Excluded** | **Descendants** | **Mapped** |
| --- | --- | --- | --- | --- | --- | --- |
| 374060 | Acute ill-defined cerebrovascular disease | Condition | SNOMED | NO | YES | NO |
| 4108356 | Cerebral infarction due to embolism of cerebral arteries | Condition | SNOMED | NO | YES | NO |
| 4110192 | Cerebral infarction due to thrombosis of cerebral arteries | Condition | SNOMED | NO | YES | NO |
| 4043731 | Infarction - precerebral | Condition | SNOMED | NO | YES | NO |

## Suicide and suicidal ideation

Note: This algorithm is based on the review by Callagan et al. [11]

Initial Event Cohort

People having any of the following: 

- a condition occurrence of Suicide and suicidal ideation^1^
  - for the first time in the person's history
- an observation of Suicide and suicidal ideation^1^
  - for the first time in the person's history

with continuous observation of at least 0 days prior and 0 days after event index date, and limit initial events to: **earliest event per person.**

Limit qualifying cohort to: **earliest event per person.**

No end date strategy selected. By default, the cohort end date will be the end of the observation period that contains the index event.

Appendix 1: Concept Set Definitions

1. Suicide and suicidal ideation

| **Concept Id** | **Concept Name** | **Domain** | **Vocabulary** | **Excluded** | **Descendants** | **Mapped** |
| --- | --- | --- | --- | --- | --- | --- |
| 439235 | Self inflicted injury | Condition | SNOMED | NO | YES | NO |
| 4181216 | Self-administered poisoning | Condition | SNOMED | NO | YES | NO |
| 444362 | Suicidal deliberate poisoning | Condition | SNOMED | NO | YES | NO |
| 4273391 | Suicidal thoughts | Condition | SNOMED | NO | YES | NO |
| 440925 | Suicide | Observation | SNOMED | NO | YES | NO |

## Tinnitus

Note: This algorithm follows Lee et al. [12]

Initial Event Cohort

People having any of the following: 

- a condition occurrence of Tinnitus^1^
  - for the first time in the person's history

with continuous observation of at least 0 days prior and 0 days after event index date, and limit initial events to: **earliest event per person.**

Limit qualifying cohort to: **earliest event per person.**

No end date strategy selected. By default, the cohort end date will be the end of the observation period that contains the index event.

Appendix 1: Concept Set Definitions

1. Tinnitus

| **Concept Id** | **Concept Name** | **Domain** | **Vocabulary** | **Excluded** | **Descendants** | **Mapped** |
| --- | --- | --- | --- | --- | --- | --- |
| 377575 | Tinnitus | Condition | SNOMED | NO | YES | NO |

## Ventricular arrhythmia and sudden cardiac death

Note: This algorithm follows the definition used by Leonard et al. [13]

Initial Event Cohort

People having any of the following: 

- a condition occurrence of Ventricular arrhythmia and sudden cardiac death^1^
  - for the first time in the person's history
  - condition type is any of: Inpatient detail - primary, Inpatient header - primary, Primary Condition, Carrier claim detail - 1st position, Carrier claim header - 1st position, Inpatient detail - 1st position, Inpatient header - 1st position, Outpatient detail - 1st position, Outpatient header - 1st position
  - visit occurrence is any of: Emergency Room Visit, Inpatient Visit

with continuous observation of at least 0 days prior and 0 days after event index date, and limit initial events to: **earliest event per person.**

Limit qualifying cohort to: **earliest event per person.**

No end date strategy selected. By default, the cohort end date will be the end of the observation period that contains the index event.

Appendix 1: Concept Set Definitions

1. Ventricular arrhythmia and sudden cardiac death

| **Concept Id** | **Concept Name** | **Domain** | **Vocabulary** | **Excluded** | **Descendants** | **Mapped** |
| --- | --- | --- | --- | --- | --- | --- |
| 321042 | Cardiac arrest | Condition | SNOMED | NO | YES | NO |
| 442289 | Death in less than 24 hours from onset of symptoms | Observation | SNOMED | NO | YES | NO |
| 441139 | Instantaneous death | Observation | SNOMED | NO | YES | NO |
| 4132309 | Sudden death | Observation | SNOMED | NO | YES | NO |
| 4185572 | Ventricular arrhythmia | Condition | SNOMED | NO | YES | NO |
| 437894 | Ventricular fibrillation | Condition | SNOMED | NO | YES | NO |
| 4103295 | Ventricular tachycardia | Condition | SNOMED | NO | YES | NO |

## Vertigo

Initial Event Cohort

People having any of the following: 

- a condition occurrence of Vertigo^1^
  - for the first time in the person's history

with continuous observation of at least 0 days prior and 0 days after event index date, and limit initial events to: **earliest event per person.**

Limit qualifying cohort to: **earliest event per person.**

No end date strategy selected. By default, the cohort end date will be the end of the observation period that contains the index event.

Appendix 1: Concept Set Definitions

1. Vertigo

| **Concept Id** | **Concept Name** | **Domain** | **Vocabulary** | **Excluded** | **Descendants** | **Mapped** |
| --- | --- | --- | --- | --- | --- | --- |
| 78162 | Peripheral vertigo | Condition | SNOMED | NO | YES | NO |
| 439383 | Vertigo | Condition | SNOMED | NO | YES | NO |
| 381035 | Vertigo of central origin | Condition | SNOMED | NO | YES | NO |

# Document S3. Consistency with gold standard from randomized controlled trials

Sertraline increases risk of diarrhea relative to comparators: Across all four databases, we consistently observe sertraline having an increased risk of diarrhea relative to most comparator treatments, with the magnitude of effect estimates ranging from 20% to 100% increased risk. In MDCR, we produce estimates for 11 comparisons with sertraline, 10 of which yield calibrated 95% confidence intervals greater than 1: nortriptyline HR=2.10 (95% CI: 1.51-2.91); psychotherapy HR=1.96 (95% CI: 1.22-3.41); fluoxetine HR=1.71 (95% CI: 1.34-2.20); sertraline HR=1.68 (95% CI: 1.44-1.97); venlafaxine HR=1.58 (95% CI: 1.35-1.86); amitriptyline HR=1.56 (95% CI: 1.10-2.37); duloxetine HR=1.33 (95% CI: 1.09-1.68); trazodone HR=1.33 (95% CI: 1.12-1.59); citalopram HR=1.31 (95% CI: 1.15-1.49); escitalopram HR=1.22 (95% CI: 1.07-1.40); and mirtazapine HR=1.16 (95% CI: 0.98-1.38). All comparisons in MDCR except psychotherapy pass all empirical diagnostics, with sufficient sample near clinical equipoise, adequate covariate balance, and empirical calibration demonstrating nominal operating characteristics. For the comparison with psychotherapy, inadequate covariate balance remains after propensity score adjustment, suggesting the potential for residual bias.

Venlafaxine increases risk of nausea relative to SSRIs: Amongst the two privately-insured populations (CCAE and Optum), we find consistent evidence of a small increased risk of nausea between new users of venlafaxine and the most prevalent SSRIs – sertraline, escitalopram, and citalopram. When comparing the risk of nausea between new users of venlafaxine and sertraline in the CCAE database, the estimated calibrated HR is 1.07 (95% CI: 0.91-1.26), and Optum returns a similar effect estimate [HR=1.10 (95% CI: 1.00-1.22)]. When comparing the risk of nausea between new users of venlafaxine and escitalopram, the estimated calibrated HR in CCAE is HR=1.12 (95% CI: 1.01-1.25) and in Optum is HR=1.12 (95% CI: 0.97-1.30). When comparing the risk of nausea between new users of venlafaxine and citalopram, the estimated calibrated HR in CCAE is HR=1.08 (95% CI: 1.00-1.18) and in Optum is HR=1.05 (95% CI: 0.95-1.18). Across all databases, comparisons between venlafaxine and each SSRI demonstrate sufficient clinical equipoise, adequate covariate balance, and empirical calibration restores nominal operating characteristics. Compared with the clinical trial results, the observational studies have a lower incidence of nausea, and are directionally consistent but had a small magnitude of effect estimate.

No difference in nausea between duloxetine and paroxetine or fluoxetine: All four databases produce estimates comparing the duloxetine with fluoxetine, all of which consistently suggested no association. In CCAE, when comparing the risk of nausea between new users of duloxetine and fluoxetine, the estimated calibrated HR is 0.93 (95% CI: 0.79-1.12). Relative to paroxetine, the estimated calibrated HR is 1.01 (95% CI: 0.85-1.19). These observational studies allow the use of large samples to bound the magnitude of any potential effect to increase confidence in any conclusion around non-inferiority.

Paroxetine higher rate of sexual dysfunction than fluoxetine and sertraline: Among clinical trials of SSRIs, paroxetine is observed to have non-significant higher rates of sexual dysfunction than fluoxetine and sertraline. Across our observational databases, paroxetine is the least commonly used SSRI. In our largest dataset (CCAE), there are <8000 new users used in each analysis. While the review compared treatments for the broadly defined ‘sexual dysfunction’ outcome, we estimate effects for a more narrowly defined diagnosis of ‘decreased libido’, and the incidence of events is lower in observational data than reported in the trials. When comparing the risk of decreased libido between new users of paroxetine and fluoxetine, the estimated calibrated HR is 1.40 (95% CI: 0.84-2.34), and in comparison with sertraline, the estimated calibrated HR is 1.39 (95% CI: 0.86-2.26).

Figure S1. Estimates for all control hypotheses before and after calibration


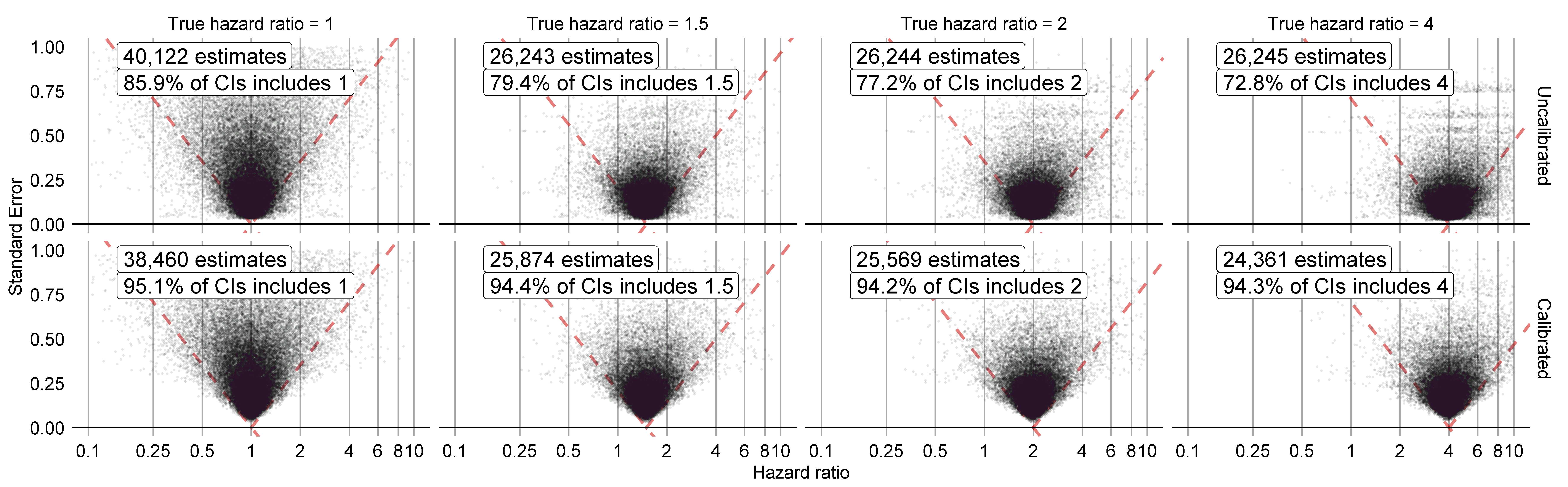


**Figure S1**. Hazard ratios and corresponding standard errors estimated through our systematic evidence generation process to four observational databases for our negative and positive controls before (top) and after (bottom) calibration. The estimates are stratified by the true hazard ratio. Note that due to limitations in sample size not all negative controls could be used to synthesize positive controls, and a small fraction of estimates could therefore not be calibrated.

# Figure S2. Leave-one-out cross-validation of calibration

To validate our confidence interval calibration procedure we use a leave-one-out cross-validation. For each negative control and the positive controls derived from that negative control, we fit systematic error models using all other controls, and compute confidence intervals for the left-out controls with a wide range of widths. We subsequently check how often the confidence intervals contained the true hazard ratio. In each fold of the cross-validation, error models are computed separately for each combination of target, comparator, and database. Figure S3 shows the coverage as a function of width of the confidence interval, stratified by the true hazard ratio.


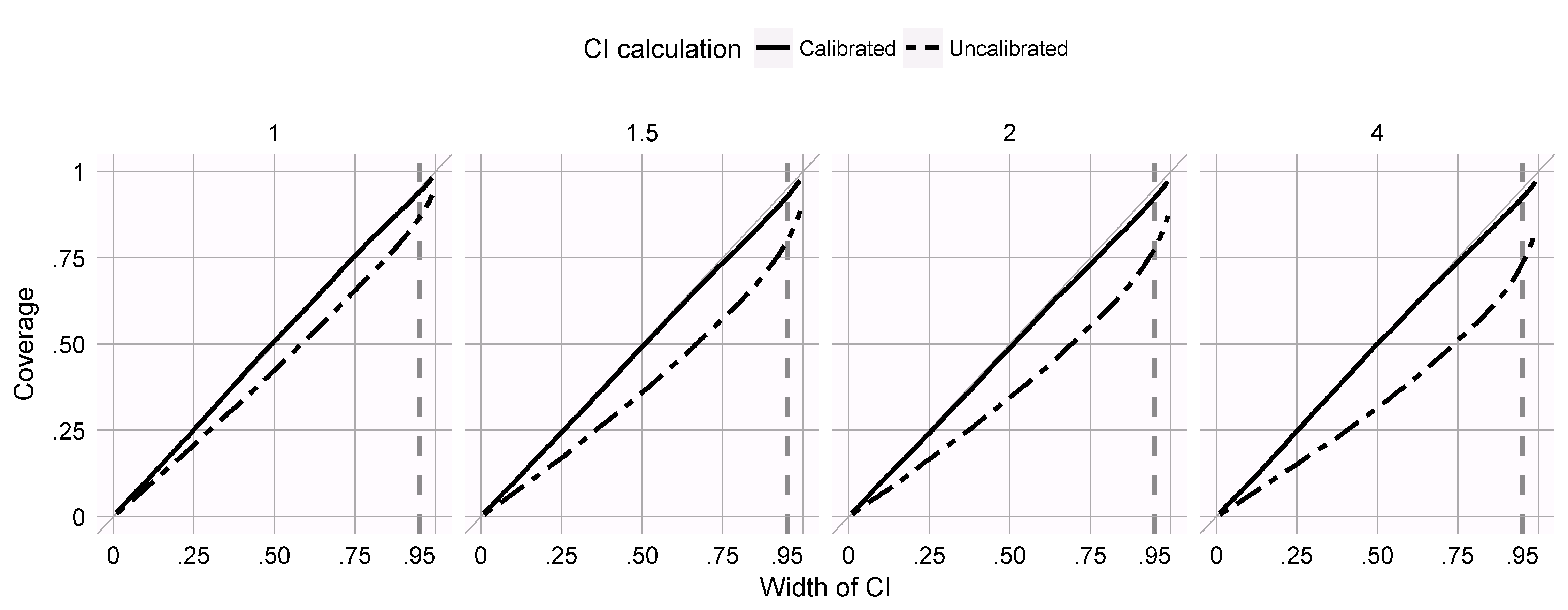


**Figure S2.** Coverage of confidence intervals, per width of the confidence interval, and stratified by true hazard ratio.

# Figure S3. Comparison of results from RCTs and our observational study


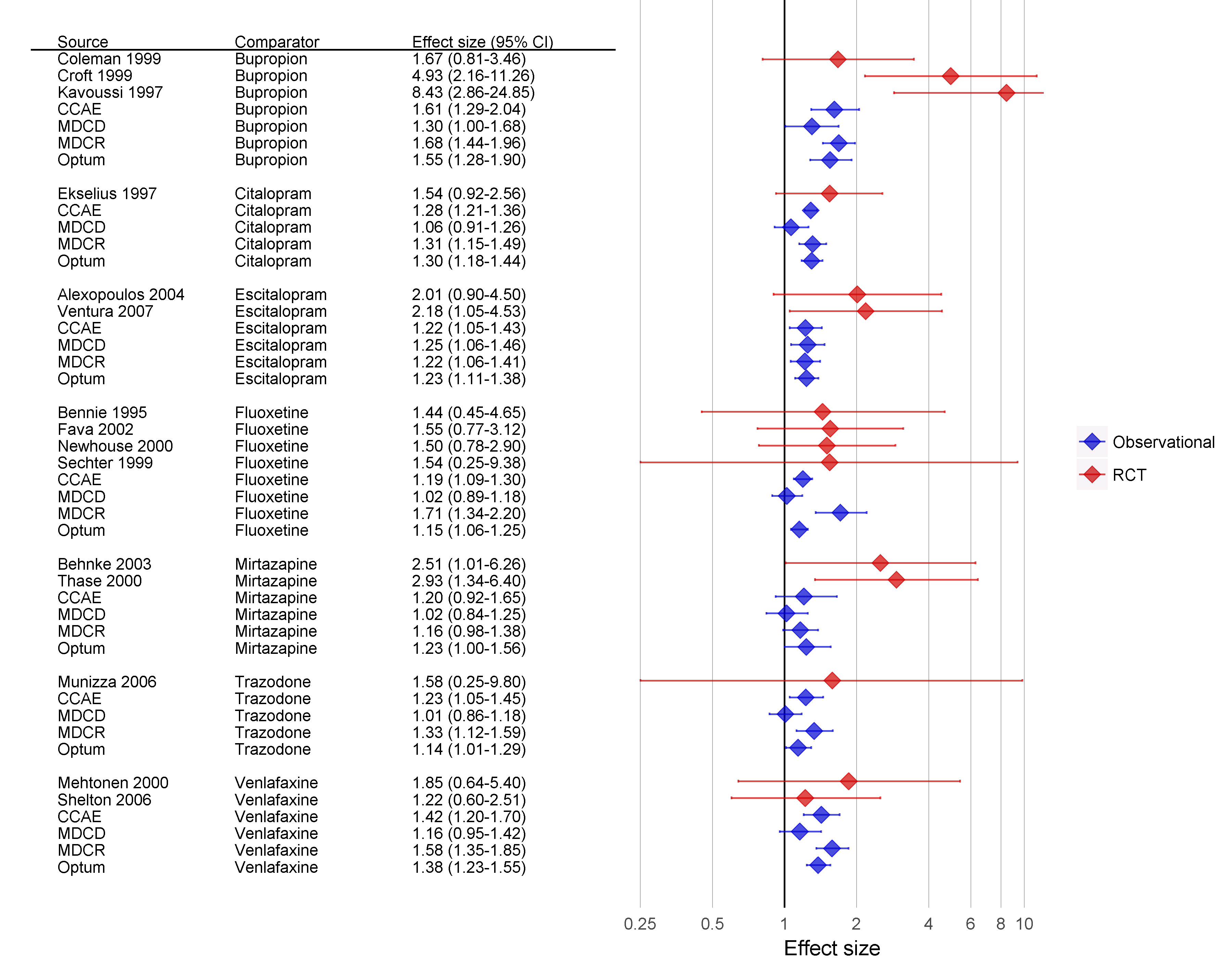


**Figure S3.** Effect size estimates on Sertraline compared to various other drugs for the outcome of diarrhea. Estimates from RCTs are those reported in Cipriani et al (2010). Observational estimates are the hazard ratios and 95% calibrated confidence intervals generated in our study.

# References

1. Overhage, J.M., et al., *Validation of a common data model for active safety surveillance research.* J Am Med Inform Assoc, 2012. **19**(1): p. 54-60.

2. Udo, R., et al., *Validity of diagnostic codes and laboratory measurements to identify patients with idiopathic acute liver injury in a hospital database.* Pharmacoepidemiol Drug Saf, 2016. **25 Suppl 1**: p. 21-8.

3. Mody, R., et al., *Prevalence and risk of developing comorbid conditions in patients with chronic constipation.* Curr Med Res Opin, 2014. **30**(12): p. 2505-13.

4. McCoy, T.H., Jr., et al., *Underreporting of Delirium in Statewide Claims Data: Implications for Clinical Care and Predictive Modeling.* Psychosomatics, 2016. **57**(5): p. 480-8.

5. Broder, M.S., et al., *Healthcare and economic impact of diarrhea in patients with carcinoid syndrome.* World J Gastroenterol, 2016. **22**(6): p. 2118-25.

6. Lanteigne, A., et al., *Serotonin-norepinephrine reuptake inhibitor and selective serotonin reuptake inhibitor use and risk of fractures: a new-user cohort study among US adults aged 50 years and older.* CNS Drugs, 2015. **29**(3): p. 245-52.

7. Wernli, K.J., et al., *Risks Associated With Anesthesia Services During Colonoscopy.* Gastroenterology, 2016. **150**(4): p. 888-94; quiz e18.

8. Lu, M.C., et al., *Higher Risk of Thyroid Disorders in Young Patients with Type 1 Diabetes: A 12-Year Nationwide, Population-Based, Retrospective Cohort Study.* PLoS One, 2016. **11**(3): p. e0152168.

9. Stein, J.D., T.S. Blachley, and D.C. Musch, *Identification of persons with incident ocular diseases using health care claims databases.* Am J Ophthalmol, 2013. **156**(6): p. 1169-1175 e3.

10. Wu, C.S., et al., *Comparative risk of seizure with use of first- and second-generation antipsychotics in patients with schizophrenia and mood disorders.* J Clin Psychiatry, 2016. **77**(5): p. e573-9.

11. Callahan, S.T., et al., *Identifying suicidal behavior among adolescents using administrative claims data.* Pharmacoepidemiol Drug Saf, 2013. **22**(7): p. 769-75.

12. Lee, C.F., et al., *Increased risk of tinnitus in patients with temporomandibular disorder: a retrospective population-based cohort study.* Eur Arch Otorhinolaryngol, 2016. **273**(1): p. 203-8.

13. Leonard, C.E., et al., *Antidepressants and the risk of sudden cardiac death and ventricular arrhythmia.* Pharmacoepidemiol Drug Saf, 2011. **20**(9): p. 903-13.
